# Minimal cells, maximal knowledge

**DOI:** 10.7554/eLife.45379

**Published:** 2019-03-12

**Authors:** Jean-Christophe Lachance, Sébastien Rodrigue, Bernhard O Palsson

**Affiliations:** 1 Département de Biologie Université de Sherbrooke Sherbrooke Canada; 2 Department of Bioengineering University of California San Diego United States; 3 Department of Pediatrics University of California San Diego United States; 4 Novo Nordisk Foundation Center for Biosustainability Technical University of Denmark Lyngby Denmark; 5 Bioinformatics and Systems Biology Program University of California San Diego Unites States

**Keywords:** systems biology, mathematical modeling, metabolism, minimal cells, gene essentiality, JCVI-syn3.0A, Other

## Abstract

Modeling all the chemical reactions that take place in a minimal cell will help us understand the fundamental interactions that power life.

**Related research article** Breuer M, Earnest EE, Merryman C, Wise KS, Sun L, Lynott MR, Hutchison CA, Smith HO, Lapek JD, Gonzalez DJ, de Crécy-Lagard V, Haas D, Hanson AD, Labhsetwar P, Glass JI, Luthey-Schulten Z. 2019. Essential metabolism for a minimal cell. *eLife* **8**:e36842. doi: 10.7554/eLife.36842

If we could map and understand every single molecular process in a cell, we would have a better grasp of the fundamental principles of life. We could ultimately use this knowledge to design and create artificial organisms. An obvious way to start this endeavor is to study minimal cells, natural or synthetic organisms that contain only the bare minimum of genetic information needed to survive. By building and studying these very simplified cells – so simple they have been described as the ‘hydrogen atoms of biology’ (Morowitz, 1984) – we may be able to dissect all the molecular mechanisms required to sustain cellular life.

The elucidation of the DNA double helix in 1953, and the subsequent cracking of the genetic code, made it possible to link molecular processes to DNA sequences (Figure 1). In turn, whole genome sequencing has revealed a collection of molecular roles encoded in the genomes of a great number of organisms, starting in 1995 with the first complete bacterial genomes (Fleischmann et al., 1995; Fraser et al., 1995), and then expanding thanks to next-generation sequencing methods (McGuire et al., 2008; Spencer, 2008). Yet, this has also showed that we do not know or can only guess the roles of many genes which are essential to life.

**Figure 1.**
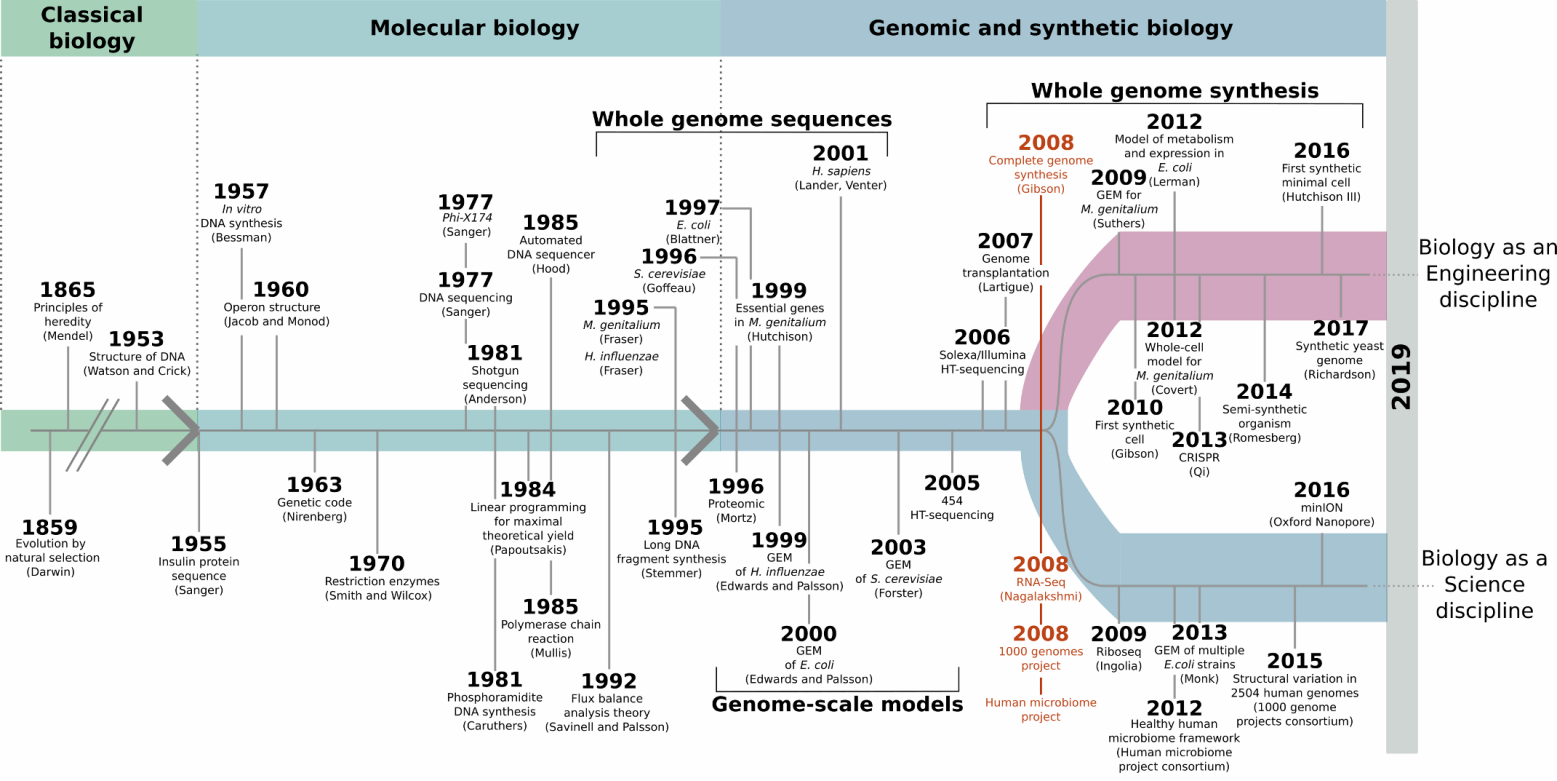
Synthetic biology and minimal cells: an historical perspective. Elucidating the DNA double helix marked the beginning of the molecular biology era, and it became possible to study molecular mechanisms that underpinned observable phenotypes. DNA sequencing methods improved, leading to whole-genome sequencing at the end of the 1990s. Methods for mathematical cell modeling were developed during the 1980s and 1990s, and computer simulations of metabolic networks (also known as genome-scale models of metabolism, or GEMs) could be reconstructed. A defining moment took place in 2008 (red), with the creation of the first artificial genome that mimicked the genetic information of *M. genitalium*, the free-living, non-synthetic organism with the smallest genome. Thanks to developments in next-generation sequencing methods, this was paired with the rise of large-scale genome sequencing ventures, such as the human microbiome and the 1000 genomes projects. Advances in whole-genome synthesis, assembly, and transplantation helped create the first cell living with an entirely synthetic genome shortly after. Taken together, these achievements marked the coming of age for synthetic biology.

In 2008, as large-scale sequencing projects were initiated, a group of scientists at the J. Craig Venter Institute (JCVI) artificially recreated the genome of a bacterium. The team made DNA fragments in the laboratory, and then used a combination of chemistry and biology techniques to assemble the pieces ‘in the right order’, using the genetic information of the *Mycoplasma genitalium* bacteria as a template (Gibson et al., 2008). This marked a significant branching point in the history of biology: while the previous decades had focused on acquiring as much knowledge as possible about natural organisms, creating a genome from scratch in a laboratory demonstrated the potential to design synthetic cells (Figure 1). This shifted synthetic biology, the field in which researchers try to build biological entities, towards an engineering discipline that could work at the scale of a genome. The same team then went on to build *Mycoplasma mycoides* JCVI-syn1.0, the first living cell with an entirely artificial chromosome (Gibson et al., 2010). In both cases, the artificial genetic information faithfully replicated that found in the wild-type bacteria.

The next goal was to piece together an artificial genome that contains only those genes that are absolutely necessary for life and growth. In 2016, after years of design and testing, the genetic information in JCVI-syn1.0 was whittled down to produce *M. mycoides* JCVI-syn3.0, which harbors the smallest genome of any free-living organism (Hutchison et al., 2016). Notably, JCVI-syn3.0 was originally reported to contain 149 genes whose roles were unknown. Since then this number has shrunk to 91, and further reducing this figure still represents the next challenge in synthetic biology (Danchin and Fang, 2016). 

Now, in eLife, Zan Luthey-Schulten and colleagues at the JCVI, the University of Illinois at Urbana-Champaign, the University of California at San Diego, and the University of Florida – including Marian Breuer as first author – report the first computational or 'in silico' model for a synthetic minimal organism (Breuer et al., 2019). The team reconstructed the complete set of chemical reactions that take place in the organism (that is, its metabolism). This effort bridges the gap between DNA sequences and molecular processes at the level of an entire biological system.

Breuer et al. performed their modeling work on *M. mycoides* JCVI-syn3.0A, a robust variation of JCVI-syn3.0 that contains 11 more genes. This was required because genome reduction involves a high number of genetic modifications, which tend to produce weaker cells that are harder to grow under laboratory conditions (Choe et al., 2019). To create their computational model, the team used the biochemical knowledge readily available for the parent strain JCVI-syn1.0 and identified the remaining candidate genes that participate in metabolism in JCVI-syn3.0A. These genes were then associated with cellular chemical reactions and, step-by-step, the entire metabolic network was modeled. This approach regroups the extensive knowledge on the metabolism of JCVI-syn3.0A in a single, highly valuable community resource that can help interrogate missing roles in the metabolic network and integrate experimental data.

Once a genome-scale model was obtained, it became possible to use it to perform computer simulations of different cellular phenotypes. Briefly, the in silico model represents the optimal metabolic state of the cell as an optimization problem on which constraints are applied. For instance, the metabolic models are constrained by the balance of reactants and products in a given chemical reaction (stoichiometry), and the conversion rates of the metabolites (flux bounds). Breuer et al. simulated the growth phenotype of JCVI-syn3.0A by optimizing for the production of cellular biomass, and then juxtaposed the predictions with real-life data, such as results from quantitative proteomics studies. In particular, they compared the genes that the model deemed essential with those highlighted when systematically mutating the genome of JCVI-syn3.0A. This revealed 30 genes that are required for survival but whose role is unknown. Understanding what these genes do is the next priority in the effort to complete the characterization of all molecular processes in a cell.

Overall, the model and experimental data generally agreed on their identification of essential genes; yet, a perfect match was not achieved, as is also the case when similar computational models are applied to natural organisms. Still, one would imagine that if this standard were within reach, it would be achieved first for minimal cells. To improve the quality of prediction, constraints that are more accurate need to be applied, and this would require additional information. For example, a completely defined media that contains only the necessary nutrients for JCVI-syn3.0A should be generated. It would also prove useful to have a precise biomass composition, that is, a detailed report of the proportion of major molecules and metabolites in the cell. Finally, many biochemical processes, such as isozymes (when enzymes with different structures catalyze the same reaction) or promiscuous reactions (when an enzyme can participate in many reactions) would need to be carefully investigated.

Such constraint-based modeling may be key to help with the generation of working genomes from square one, and in this regard, the model generated by Breuer et al. is the first of many steps to perfectly mirror a synthetic cell in silico. Next, the simulation could be expanded beyond metabolism to include other sets of biological processes, such as the gene expression machinery. This would help identify key constraints and trade-offs that cells must deal with in the struggle for life. In turn, these constraints could become the framework required to artificially design increasingly complex organisms, much like the hydrogen atom paved the way to understanding the behavior of more complex elements.
